# The olfactory bulb theta rhythm follows all frequencies of diaphragmatic respiration in the freely behaving rat

**DOI:** 10.3389/fnbeh.2014.00214

**Published:** 2014-06-11

**Authors:** Daniel Rojas-Líbano, Donald E. Frederick, José I. Egaña, Leslie M. Kay

**Affiliations:** ^1^Committee on Neurobiology, The University of ChicagoChicago, IL, USA; ^2^Institute for Mind and Biology, The University of ChicagoChicago, IL, USA; ^3^Department of Psychology, The University of ChicagoChicago, IL, USA; ^4^Departamento de Anestesiología y Reanimación, Facultad de Medicina, Universidad de ChileSantiago, Chile

**Keywords:** local field potential, olfactory bulb, respiration, rat, theta rhythm

## Abstract

Sensory-motor relationships are part of the normal operation of sensory systems. Sensing occurs in the context of active sensor movement, which in turn influences sensory processing. We address such a process in the rat olfactory system. Through recordings of the diaphragm electromyogram (EMG), we monitored the motor output of the respiratory circuit involved in sniffing behavior, simultaneously with the local field potential (LFP) of the olfactory bulb (OB) in rats moving freely in a familiar environment, where they display a wide range of respiratory frequencies. We show that the OB LFP represents the sniff cycle with high reliability at every sniff frequency and can therefore be used to study the neural representation of motor drive in a sensory cortex.

## Introduction

Whenever the performance of a sensory system becomes crucial for an animal, it is always in the context of interaction with its immediate environment, such as when searching for food, hunting, escaping, mating, or simply exploring its surroundings. Animals execute these tasks by actively manipulating sensory stimuli through motor activities that sample physical features in time and space. Well known examples are eye movements and finger movements in primates, whisking in rodents, and sniffing in mammals. Active stimulus manipulation facilitates feature selection from complex stimuli during learning processes, ongoing adaptation to the task at hand and optimization of stimulus discrimination (Mitchinson et al., 2007; Rojas-Líbano and Kay, 2012). Neural circuits implicated in the different motor sampling modalities comprise almost all brain systems, display profuse interconnection with sensory areas and underlie higher-level cognitive functions (Liversedge and Findlay, 2000; Diamond et al., 2008; Wachowiak, 2011).

In olfaction, stimuli must be carried from external air to the sensory epithelium inside the nose. Mammals accomplish this through sniffing, a voluntary modification of regular breathing. Sniffing motor patterns have effects on the temporal structure of sensory input (Carey et al., 2009), subsequent brain processing (Carey and Wachowiak, 2011) and gain modulation of incoming sensory signals (Verhagen et al., 2007; Courtiol et al., 2011a; Rosero and Aylwin, 2011; Esclassan et al., 2012). However, there are no systematic studies of the basal conditions of the system and its internal neural representation, close to its ecological mode of operation. For example, rodents navigate through an environment constantly sensing and learning, mainly through whisking and sniffing. This allows them to gauge object distances, track entities of interest, and adapt their displacement accordingly and generally to learn about their immediate environments.

In mammals, it is hypothesized that air entry on each respiration cycle causes either a mechanical or odor signal, or both, detected by olfactory sensory neurons in the nose (Ueki and Domino, 1961; Onoda and Mori, 1980; Grosmaitre et al., 2007). This, in turn, produces a strong local field potential (LFP) oscillation in the olfactory bulb (OB) that represents the sniff cycle in voltage change over time. Early efforts by Walter Freeman combining modeling and recording hypothesized that the transient coordinated volley of impulses arriving to the OB with each inspiration would modify gain parameters of the OB neuronal network. This in turn would cause a transient increase in OB activity, reflected in LFP oscillations at the respiratory frequency, a phase-locked burst of gamma activity, and an increase in single neuron activities (Freeman, 1975). Empirical evidence for this relationship comes from anesthetized preparations (Adrian, 1950; Fontanini et al., 2003) or from short-time example traces in awake animals (Freeman, 1976; Courtiol et al., 2011b), and most of the time in the context of odorant stimulation. The normal activity range within which this respiratory-neural correlation is valid is therefore unknown, as is whether it holds in conditions of regular exploratory behavior, with non-specific odorant stimulation.

Given this lack of systematic evidence, we wanted to address at least two experimental questions. Does the strong coupling between respiration and the OB LFP hold for the continually-adapting sniffing activity displayed in free exploratory behavior? Does this relationship change with the animal's behavior?

We addressed these questions in freely behaving rats using a task-free paradigm to encourage the rats to use a wide range of exploratory respiratory behaviors. On the motor side, we monitored the diaphragm, the main muscle target of the respiration network. Simultaneously we monitored the sensory side by recording LFPs from the olfactory bulb. We found that the relationship between motor sampling behavior and sensory activity is consistent across all respiratory frequencies without specific odorant stimulation other than the normal olfactory environment. We also found that several features of respiration are present in and therefore can be reliably extracted from OB oscillations, and that different behavioral states modulate the relationship. Our data show an ongoing sensory-motor coupling across a range of normal behaviors. In a situation like this, when there are no clear-cut, experimenter-controlled stimulus delivery times, and the animal is moving freely, our results also illustrate the usefulness of investigating sensory responses using the temporal frame of the relevant motor sampling activity.

## Materials and methods

### Subjects

Five adult male Sprague Dawley rats were used in the experiments (Harlan). Of these, two rats also participated in an olfactory discrimination protocol published elsewhere (Rojas-Líbano and Kay, 2012). All rats were housed individually in standard clear polycarbonate home cages with filter tops and maintained on a 14/10 h light/dark cycle [lights on at 8:00 AM central standard time (CST)]. All recording sessions were performed during the light phase, between 9:00 AM and 5:00 PM CST. All experimental procedures were done with approval and oversight by the University of Chicago Institutional Animal Care and Use Committee, according to Association for Assessment and Accreditation of Laboratory Animal Care guidelines.

### Electrode fabrication

LFP electrodes were made using 100 μm diameter, formvar-coated stainless steel wire (304 HFV, California Fine Wire, Grover Beach, CA). On each recording site were implanted two electrodes previously glued together longitudinally, with an inter-tip separation of ~0.5 mm. Impedance of LFP electrodes was in the range of 50–100 kΩ.

Ground and reference electrodes were made using 250 μm diameter, polyimide-coated stainless steel wire (304 H-ML, California Fine Wire, Grover Beach, CA). A ~10 cm long piece was cut and ~2–3 cm of insulation removed at one end, which was wrapped around a small stainless steel screw (MPX-080-2P, Size:#0−80 × 1/8", Pan head style, Small Parts Inc., Miami Lakes, FL). At the other end, ~0.5 cm of insulation was removed and the exposed wire was crimped to a gold connector.

EMG electrodes were made following the design of Shafford et al., designed originally for recording of the diaphragm EMG in awake rabbits (Shafford et al., 2006). A full description of the fabrication can be found elsewhere (Rojas-Líbano and Kay, 2012). Briefly, the EMG recording electrode was made using a piece of 250 μm diameter stainless steel wire forming a coil of length 1–2 mm. This piece was attached to a flexible, multistranded stainless steel wire (AS 155-30, Cooner Wire, Chatsworth, CA).

Prior to surgery, EMG electrodes and their wires were disinfected in a solution of benzalkonium chloride (diluted 1:750).

### Surgery

Rats were anesthetized initially using a Ketamine-Xylazine-Acepromazine cocktail injected subcutaneously. Anesthesia during surgery was maintained using Pentobarbital sodium solution administered intraperitoneally. Rodent aseptic surgery guidelines were followed for all surgical procedures (Cunliffe-Beamer, 1993). A midline incision was done to gain access to the abdominal cavity. EMG electrodes were held by the heat shrink tubing segment using a hemostat and inserted into the diaphragm, perpendicular to the diaphragm surface. Three EMG leads were implanted in each rat, at the right costal diaphragm. The flexible wire was passed through the abdominal muscles, 2–3 cm lateral to the midline incision, and knots were tied with the same wire cable on both sides of the abdominal muscle to prevent excess wire movement. The abdominal muscle incision was sutured using absorbable polyglycolic acid suture (Surgical Specialties Co, Reading, PA). Electrode wires were tunneled under the skin and exteriorized at the back of the head. The abdominal skin incision was sutured with non-absorbable polyamide suture (Henry Schein, Melville, NY). Placement of diaphragm electrodes took ~2.5 h from incision to suturing.

After placement of EMG electrodes, rats were placed in a stereotaxic frame. Electrodes were positioned using stereotaxic coordinates with respect to Bregma, in the OB (8.5 mm anterior, 1.5 mm lateral) and the PC (0.5 mm anterior, 3 mm lateral). The reference and ground electrodes were screwed into the skull. All gold connectors, from EMG, LFP and ground/reference electrodes, were inserted into a threaded round nine-pin socket (GS09SKT-220; Ginder Scientific) and fixed onto the rat's head with dental acrylic.

Postsurgical analgesia was provided via subcutaneous injection of buprenorphine (Buprenex®). Recording sessions were performed 15 days after surgery at the earliest.

### Experimental protocols and data acquisition

For the recording sessions, rats were connected to the recording system and then placed back in their home cages, using a modified cage top to pass the cable through it. The arrangement allowed the rat to move freely in the cage. Recordings lasted 10–25 min.

Electrophysiological data were acquired using a Cheetah-32 (Neuralynx, Bozeman, MT) amplifier system of 2.5 MΩ input impedance, and a unity gain headstage preamplifier (HS-18-CNR, Neuralynx). During acquisition, signals were amplified (2000×) and filtered online with an analog Butterworth filter (Rolloff 12 DB per Octave). LFP channels were filtered between 1 and 200 Hz or between 1 and 475 Hz. EMG raw channels were filtered between 1 and 3000 Hz and EMG bipolar channels were filtered between 300 and 3000 Hz. In some sessions, all channels were sampled at 1892 Hz. In other sessions, LFP channels were sampled at 2000 Hz and EMG channels at 8000 Hz.

### Data analysis

All data analysis was done offline using MATLAB®. Code was written to store and process the data. Neuralynx MATLAB function Nlx2MatCSC was used to import the data into MATLAB.

#### EMG data processing

In order to extract the respiratory parameters (i.e., inhalation duration, tidal volume, cycle duration, inhalation strength), the EMG signal was processed. Full details of this processing are described elsewhere (Rojas-Líbano and Kay, 2012). First, the signal was rectified by taking its absolute value. Second, a moving mean filter of the signal was calculated using a window of 40 ms, with a step size of 1 sample, to produce the rectified and smoothed EMG (rsEMG). Each point of the rsEMG was calculated according to the formula:

$$y\left(t\right)=\frac{1}{\text{2}w\text{+1}}\sum_{t\text{-}w}^{t\text{+}w}x(t)$$

where *x*(*t*) is the original rectified EMG, 2*w* + 1 is the dimension of the window and *y*(*t*) is rsEMG, with *t* representing the index of the sample at any given time.

#### Inhalation-detection algorithm

An algorithm was written in MATLAB to automate the detection of inhalation episodes. The timing of inhalation (start and end points) is the only parameter required to calculate all the respiratory timing variables. The algorithm operated on the rsEMG signal and compared it with a moving threshold signal (moving-mean filter applied to rsEMG with window of 80 ms). In a first pass, any points on the rsEMG above the threshold were kept and the rest were made equal to zero. Then a collection of indices was obtained by searching for all the transitions from zero to positive (inhalation start), and from positive to zero (inhalation end). In a second pass, a minimum inhalation duration (30 ms) value was defined and all the inhalation events shorter than the minimum were removed. In a third and final pass, a minimum exhalation duration (10 ms) value was defined and all shorter events were removed. The collection of indices was used then in the original rsEMG signal to determine all respiratory variables. All inhalation episodes detected by the algorithm were visually inspected.

#### Spectral analysis

Spectral analysis was carried out with open-source Chronux algorithms (http://chronux.org). The multitaper method was used to estimate frequency spectra. Theory for multitaper estimates can be found in several articles and books (Thomson, 1982, 2001; Percival and Walden, 1993). In the multitaper procedure, a time-bandwidth *NW* product is defined, where *N* is the number of samples of the electrophysiological time series and *W* is the bandwidth of interest. Then multiple data tapers are selected and computed. The tapers correspond to a set of functions called discrete prolate spheroidal, or Slepian, sequences (Slepian, 1978), and usually the number of tapers *K* is selected to be not larger than 2*NW* − 1, with *NW* ≈ 4–6 a typical choice. Spectrum estimates are computed by taking the discrete Fourier transform of the time series multiplied by the selected tapers and averaging over tapers (Thomson, 1982).

Spectra estimates *Ŝ* were calculated as the average over *K* tapers as

$$\hat{S}\left(f\right)=\frac{1}{N}\frac{1}{K}\sum_{k\;=\;1}^{K}{\tilde{V}}^{\left(n,k\right)}\cdot {\tilde{V}}^{*(n,k)}$$

where ^*^ means complex conjugate and Ṽ^(*n*, *k*)^ is the discrete-time Fourier transform of the product between the time series *x*(*t*) and the *k*-th taper *w*^(*k*)^(*t*):

$${\tilde{V}}^{\left(n,k\right)}=\sum_{t\;=\;0}^{T}e^{i\text{2}\pi ft}w^{\left(k\right)}\left(t\right)\;V^{\left(n\right)}(t)\;\cdot \;t_{S}$$

where *f* represents frequency in Hz, *t* represents time and *T* is the duration of the time series in seconds (i.e., *T* = *N* · *t*_*S*_, with *t*_*S*_ being the sampling interval). Spectrograms were constructed by plotting spectral power during a series of overlapping constant-width time windows (overlap: 0.1 s; width: 0.4 s). Spectral coherence *C* between two signals (e.g., between rsEMG and LFP) was computed as

$$C\left(f\right)=\frac{{\hat{S}}_{12}(f)}{\sqrt{{\hat{S}}_{1}\left(f\right){\hat{S}}_{2}(f)}}$$

where *Ŝ*_12_(*f*)corresponds to the cross-spectrum calculated from the fast-Fourier transforms (FFTs) of the time-tapered vector signals, and *Ŝ*_1_(*f*), *Ŝ*_2_(*f*) correspond to the individual power spectra of the two signals. Confidence limits (95%) were estimated for coherence magnitude by a jackknife procedure. Coherencegrams were constructed by plotting spectral coherence during a series of overlapping constant-width time windows (overlap: 2 s; width: 0.3 s).

#### LFP polarity and LFP phase calculations

**Figure 1:**
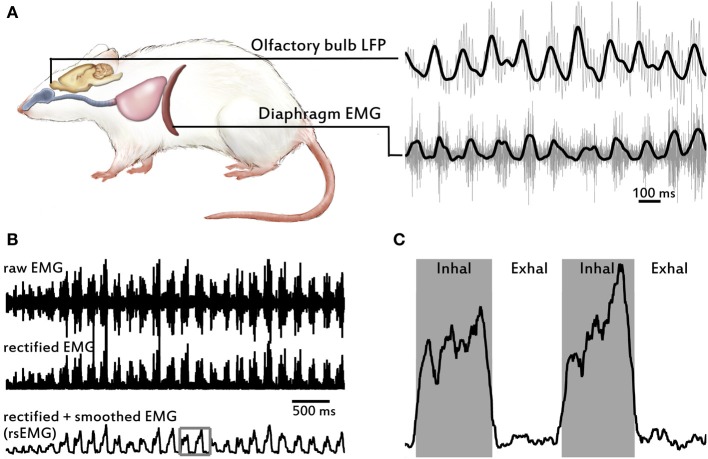
**Overview of signals recorded and EMG processing to obtain respiratory variables**. **(A)** Drawing of a rat showing the two structures and signals recorded: the local field potential (LFP) from the olfactory bulb (OB) in the brain (top trace), and the electromyogram (EMG) from the diaphragm (bottom trace). Respiratory airways from the nose to the lungs are also depicted. Gray traces correspond to raw signals and black traces to low-pass filtered signals. **(B)** Example EMG traces showing signal processing, sequentially from top to bottom. The EMG raw signal is positively rectified and then is smoothed using a moving-mean filter to produce the rsEMG. **(C)** Enlarged image of square in bottom trace in **(B)**, showing example of performance of the inhalation-detection algorithm.

Our LFP recordings from the OB were made with electrodes penetrating perpendicular to the dorsal surface of the bulb. In these conditions, electrode tips ended either dorsal (D) or ventral (V) to the mitral cell layer. Recordings in D and V positions have almost identical shape but inverted polarity, since the layered architecture of the bulb forms an electrical dipole with zero isopotential surface near the mitral cell layer (Freeman, 1972). For purposes of the analysis, all LFP recordings were transformed off-line to the same polarity, by multiplying the time series by −1 where appropriate. The polarity chosen was the polarity of D recordings, i.e., where the characteristic gamma bursts occur in the falling phase of the Theta-LFP, as shown in Figure 1A. In this orientation, the end or peak of inhalation is coincident with the theta positive peak, and exhalation occurs on the downward trajectory, ending with the theta trough.

After polarity correction, we first low-pass filtered the raw LFP at 15 Hz to calculate theta-LFP phase. Then we calculated the instantaneous phase of the filtered signal using the discrete Hilbert transform, from the MATLAB Signal Processing Toolbox. We considered as the start of the theta cycle the point of phase = −π of the instantaneous phase time series, i.e., the local minima of the theta-LFP, and the point of phase = π the end of the theta cycle (i.e., the next local minimum of the theta-LFP after cycle start).

#### LFP Phase-Amplitude coupling measure

We calculated a measure of phase-amplitude coupling to study the relationship between the phase of slow (1–15 Hz) oscillations and the amplitude of fast (40–100 Hz) oscillations in the OB LFP. Our measure was based on the methods described by Tort and colleagues in several articles (Tort et al., 2009, 2010). Briefly, we first filtered the raw LFP signal to obtain two time series containing separately the slow and fast oscillations. Then we calculated the instantaneous phase for the slow oscillation and the amplitude envelope for the fast oscillation by means of the Hilbert transform, obtaining two new time series containing the phase and amplitude measures. Out of these we calculated two estimates of phase-amplitude coupling. By binning the phase, we produced histograms of amplitude distribution over the phase. And by measuring the difference between this distribution from a uniform distribution, we also calculated a Modulation Index (MI) for the phase-amplitude coupling (Tort et al., 2010).

## Results

Relationships between respiratory behavior and what is termed the respiratory rhythm in the OB LFP have been studied only within a narrow range of frequencies. In order to study this relationship over a wide range of respiratory frequencies we used simple free behavior in the rats' home cage. Our previous research showed a tightly defined set of respiratory behaviors during odor discrimination (Rojas-Líbano and Kay, 2012), and we have observed that OB LFP properties are highly variable dependent on behavior (Kay, 2003). We recorded from chronically implanted rats during free behavior in their home cages, using a modified cage top to allow for recording. We simultaneously acquired the olfactory bulb LFP and the diaphragm EMG signals to observe the sensory and motor activities of the animals while they explored (Figure 1). Cages did not contain specific odorous stimuli, but only objects usually present: feces, bedding, and food pieces. In our experience, this odorous environment produces a full range of sniffing behaviors.

### General description of respiratory patterns

**Figure 2:**
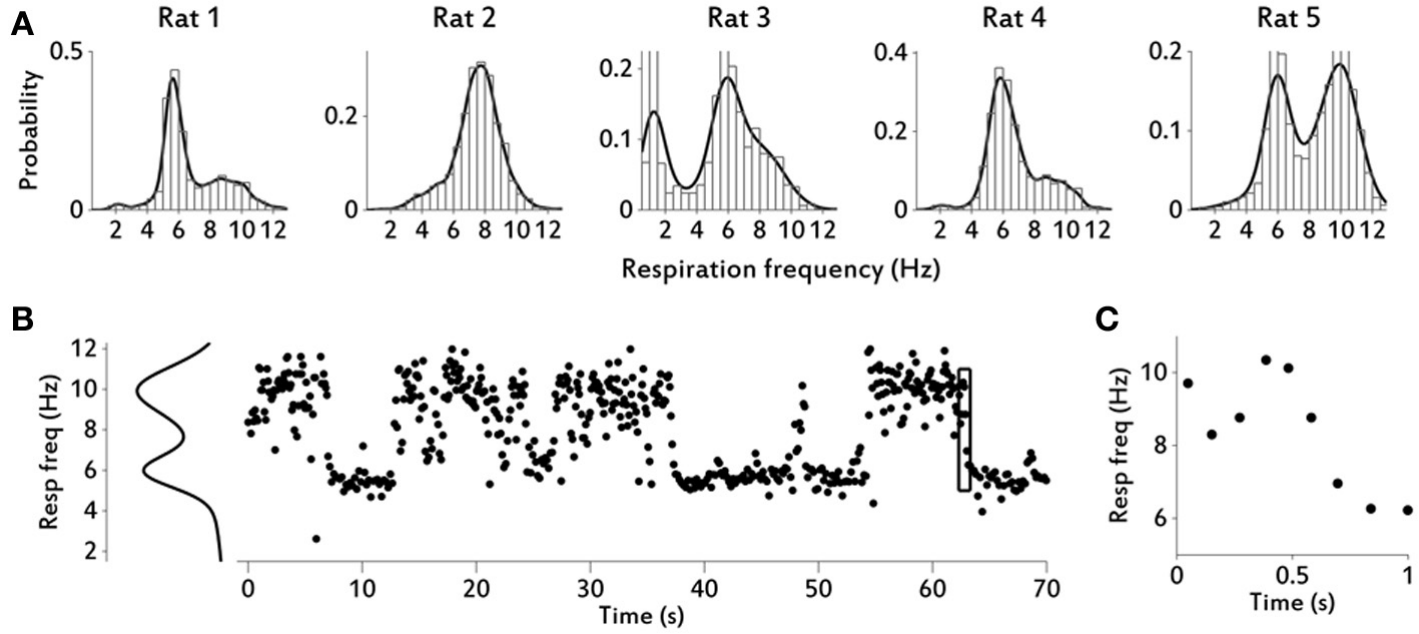
**Respiratory frequency distributions and variability over time**. **(A)** Respiratory frequency distributions for all rats. Numbers of respiratory cycles per rat are shown in Table 1. Rats 3 and 5 showed behavioral periods of quietness, almost to the point of falling asleep, especially Rat 3. In these periods, respiratory frequency decreased considerably. **(B)** Example of respiratory frequency over time during 70 s, for rat 5. Distribution fit is shown on the left. For each respiratory cycle the time assigned corresponds to time of inhalation start. **(C)** Enlarged image of section marked with a rectangle in **(B)**, to show that changes in respiratory frequencies can occur rapidly (e.g., changes of 4–6 Hz in magnitude occur in 1–2 cycles).

**Figure 3:**
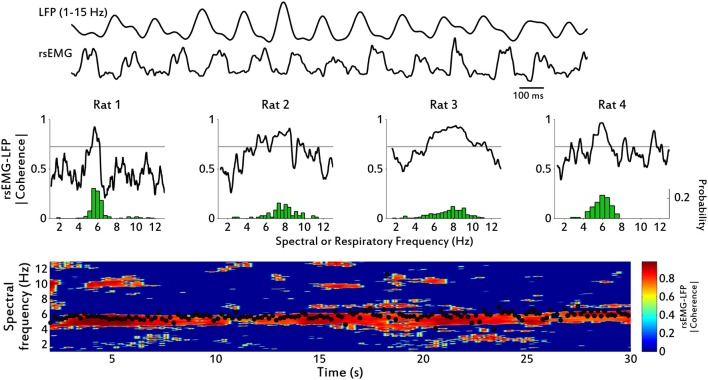
**Spectral coherence between diaphragm EMG and olfactory bulb LFP. Top:** example traces of filtered (1–15 Hz) OB LFP and rsEMG, from Rat 4. **Middle:** spectral coherence magnitude between rsEMG and low-pass filtered OB LFP for each of the four rats from which we collected both LFP and EMG signals. Dotted line marks the confidence level above which values are assumed to be significantly coherent. Note that coherent activity occurs precisely at the range of respiratory frequencies, which distribution is shown underneath in green. **Bottom:** time-frequency chart showing spectral coherence between rsEMG and LFP, for Rat 2. Coherence spectra were calculated using a 4-s moving window, stepped by 200 ms, using 7 tapers. Black dots correspond to instantaneous respiratory frequency calculated from the diaphragm rsEMG.

**Figure 4:**
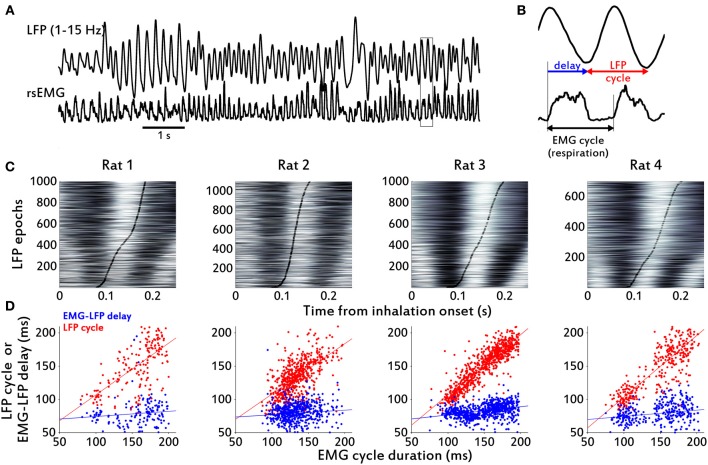
**Cycle-by-cycle agreement between diaphragmatic respiration and OB LFP. (A)** Filtered LFP (1–15 Hz) and rsEMG example traces from Rat 1. **(B)** Enlarged plot from section marked with a rectangle in **(A)**, to show the delay between EMG cycle onset and LFP cycle onset, and the LFP cycle. **(C)** Raster plots of peri-inhalation LFP epochs, sorted by respiration cycle duration (shortest at bottom; white represents values above zero). Black thick lines mark the end of the respiratory cycle estimated from the EMG for each epoch. Note that the end of the EMG cycle precedes the end of the LFP cycle with a small apparent drift as cycle length increases. **(D)** Scatter plots of cycle durations. The durations of respiratory cycles, as determined from the rsEMG signal, were plotted against the duration of the corresponding LFP cycles. Blue dots correspond to the duration of the EMG-LFP delay and red dots to the duration of the complete LFP cycles. The lines are linear fits from the regressions described in the text. Although the correlation coefficient between delay and cycle duration is very small, the small apparent drift in delay from **(C)** is also present in the non-zero slope of the line fit.

**Figure 5:**
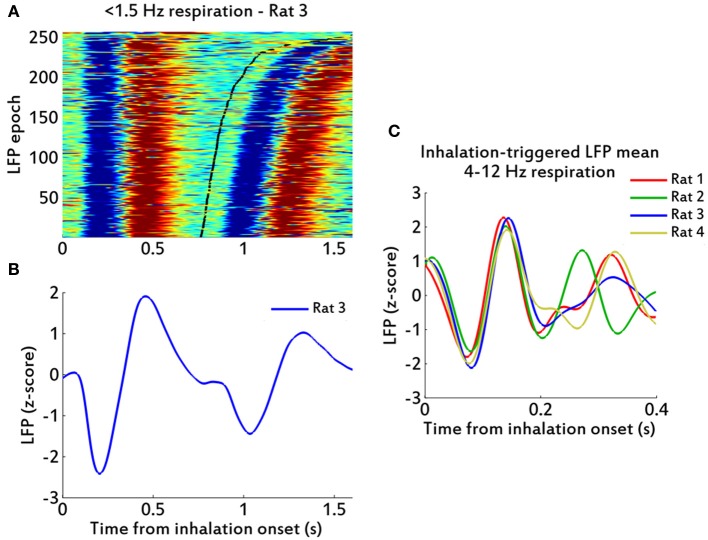
**LFP oscillations in low and high frequency respirations. (A)** Raster plot of peri-inhalation LFP epochs for rat 3, for slow (<1.5 Hz) cycles, sorted by respiration cycle duration (shortest at bottom; bottom axis in seconds). Black line marks the ends of the respiratory cycles. *Red color* indicates values greater than zero. **(B)** Inhalation-triggered LFP mean for data in **(A)**. **(C)** Inhalation-triggered LFP means for data of respiratory frequencies between (4–12 Hz), showing one trace per rat.

**Figure 6:**
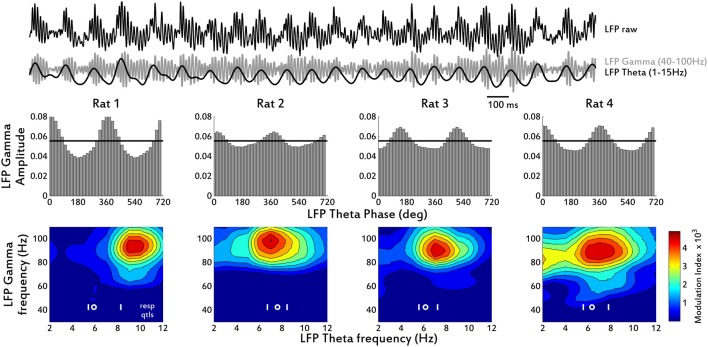
**Phase-Amplitude Coupling (PAC) measures for the OB LFP. Top:** example trace showing raw LFP and its Gamma (40–100 Hz) and Theta (1–15 Hz) filtered versions, from Rat 1. **Middle row:** mean LFP Gamma amplitude distribution over LFP Theta phase bins. For clarity, two cycles are shown (1 cycle = 360°). Black horizontal line shows the expected amplitude values for a uniform distribution (i.e., no modulation). **Bottom row:** contour plots of Modulation Index (MI) for all pairs of Gamma and Theta LFP frequencies. The data are dominated in the gamma band by high frequency activity, so little coupling is shown for lower gamma frequencies. Respiration frequency distributions from EMG data are shown as the 25th and 75th quartiles (white vertical lines) and the median (white circle).

**Figure 7:**
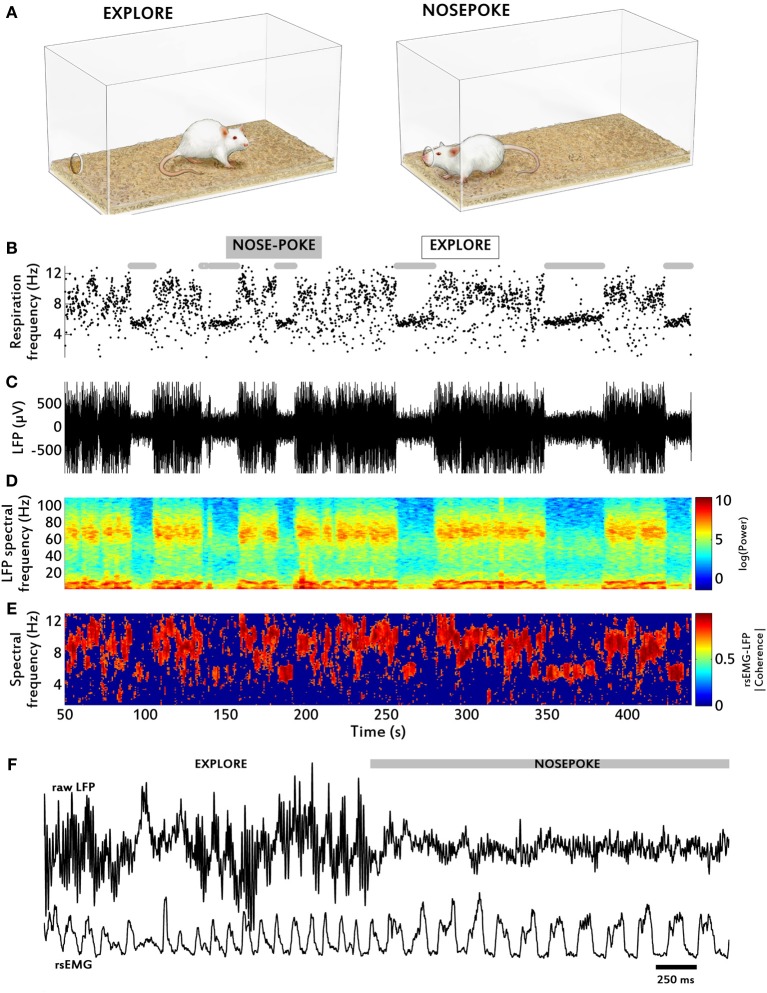
**Behavioral modulations of the sensory-motor coupling**. **(A)** Rats displayed two main behaviors: remaining still and poking the nose at a hole in the cage wall (“NOSEPOKE”) or actively exploring the cage (“EXPLORE”). **(B)** Respiratory frequency time series, calculated from diaphragm EMG; note the large variance in frequency during exploratory periods relative to nose-poke periods (marked by gray bars). **(C)** Raw LFP time series. Note the much larger amplitudes, which represent primarily theta activity at this time scale, during exploration as compared to nose-poke. **(D)** Time-frequency chart of raw LFP spectral power. During the large amplitude LFP periods **(C)** both gamma and theta power are dramatically increased relative to nose-poke. **(E)** Time-frequency chart of LFP-rsEMG spectral coherence. The magnitude of coherence does not change across frequencies in the two conditions. **(F)** Example traces of raw LFP and rsEMG before and after a transition between both behavioral modes.

We used the processed diaphragm signal, i.e., the rsEMG, to extract the relevant respiratory variables (see Materials and Methods and Figure 1). Table 1 shows central tendency and variability measures for all rats and variables. We show a total of five rats with diaphragm (respiratory) data, one of which (rat 5) did not have LFP data and therefore is not shown in figures with LFP results (Figures 2–7). Rats displayed a continuously varying respiratory frequency, consistent with free behavior, ranging approximately from 2 to 12 Hz, and could switch between frequencies rapidly, in 1–2 respiratory cycles (see Figure 2). While the entire waking respiratory range is represented in the data, all rats spent the vast majority of their time during the recorded epochs engaging in sniffing behavior (5–10 Hz), which was the object of this study. Only one rat (rat 3) produced low respiratory frequencies for a large amount of the time, and this rat had a lower mean frequency and a much larger variance in frequency than the other four rats (see Table 1). As we have previously shown for odorant sampling periods (Rojas-Líbano and Kay, 2012), the diaphragm signal allows us to monitor sniffing activity, and we extend it now to the spontaneously exhibited respiratory range of freely behaving rats, without interfering with airflow at the nasal cavity.

**Table 1:** **Means and standard deviations of respiratory parameters, and number of respiratory cycles for each rat, calculated from diaphragm rsEMG**.

	**Rat 1**	**Rat 2**	**Rat 3**	**Rat 4**	**Rat 5**
Respiratory frequency (Hz)	6.78 ± 1.99	7.47 ± 1.57	5.22 ± 2.78	6.79 ± 1.83	8.19 ± 2.23
Inhalation duration (ms)	94.66 ± 43.86	86.33 ± 46.80	241.06 ± 267.58	95.38 ± 42.76	70.56 ± 44.47
Exhalation duration (ms)	67.36 ± 25.37	56.03 ± 15.29	95.82 ± 78.21	64.79 ± 36.55	64.10 ± 20.74
n cycles	1308	1339	1570	772	2896

### Relationship between diaphragmatic respiration and the olfactory bulb LFP

Central representation of the respiratory event in cortical signals allows us to infer how much information about the motor drive is present in the sensory signal. In this case the motor activity (inhalation, exhalation) produces changes in the external immediate environment (airflow and pressure changes in nasal cavity), which result in activation of the sensory neurons and the corresponding brain structure. We therefore assess the strength of sensorimotor coupling between the OB, a cortical area that receives the sensory signal from the olfactory nerve, and the diaphragm, the muscle driven by the output of the neuronal respiratory center (Feldman et al., 2013). We first consider whether there is a general association at all respiratory frequencies associated with many types of behaviors between diaphragmatic EMG activity and the LFP of neuronal populations in the OB. In the frequency domain, we observe a strong relationship between the signals, with high spectral coherence precisely within the range of respiration exhibited by the rats (see Figure 3). For each rat we calculated the spectral frequency of maximum coherence (fmaxC) and the median respiratory frequency (medianRF), and the values obtained where (in Hz): Rat 1, fmaxC = 5.67, medianRF = 5.85; Rat2, fmaxC = 8.46, medianRF = 7.84; Rat 3, fmaxC = 8.26, medianRF = 7.8; Rat 4, fmaxC = 5.82, medianRF = 5.88. This coupling is sustained over time, as shown by spectral coherence in a time-frequency chart (Figure 3).

We also analyzed LFP epochs surrounding the diaphragmatic respiration cycle using the motor period as a temporal frame to study the sensory signal. Figure 4C shows these data in raster plots of LFP segments, sorted by respiratory cycle duration. This sorting, based exclusively on EMG data, also produced a sorting of the LFP epochs, reflecting that the temporal structure of respiratory cycles was conserved in the LFP across all cycle durations. Inspection of these rasters suggests a correlation between the respiratory cycle duration (vertical black curves in rasters) and LFP cycles starting at approximately 80 ms after inhalation onset or a constant delay between the start of respiration in the diaphragm and arrival of signal at the olfactory bulb (Figure 4B). We estimated this delay in all the rats by extracting the timepoints of the relevant LFP cycles. We used two approaches to extract cycles from the LFP time series. First, we calculated the maxima and minima from the low pass-filtered LFP (1–15 Hz) and defined one cycle to be the LFP epoch going from one minimum to the next. We also calculated the instantaneous phase of the LFP using the Hilbert transform, and defined one cycle to be the LFP epoch going between points of instantaneous phase = −π to phase = π. We obtained identical results with both techniques.

All the rats displayed a relatively constant delay between inhalation onset at the diaphragm and the start of a new LFP theta cycle in the OB (Figure 4D, blue dots). Mean and standard deviations for the delay were: Rat 1, 78.5 ± 34.1 ms; Rat 2, 79.2 ± 15.3 ms; Rat 3, 82.6 ± 12.1 ms; Rat 4, 78.8 ± 15.2 ms; Sample mean, 79.8 ± 1.9 ms. Consistent with a delay independent of respiration duration, in the linear least-squares model (LFP cycle duration) = β_0_ + β_1_. (EMG cycle duration) we found low Pearson's product-moment correlation coefficients and regression coefficients when we tried to correlate the EMG cycle duration with the simultaneously measured (i.e., exactly corresponding segment in time) LFP cycle: Rat 1, β_1_ = 0.077, β_0_ = 0.066, *r* = 0.0679; Rat 2, β_1_ = 0.069, β_0_ = 0.07, *r* = 0.095; Rat 3, β_1_ = 0.12, β_0_ = 0.064, *r* = 0.29; Rat 4, β_1_ = 0.094, β_0_ = 0.065, *r* = 0.21. (see Figure 4D, blue-dots scatter plots).

Conversely, when we assessed the correlation between respiration duration of individual cycles in the diaphragm and the duration of the LFP cycle shifted in time (i.e., measured from the start of a new LFP cycle and not exactly simultaneous with EMG cycle), we found that data in all rats were consistent with a cycle-by-cycle representation of diaphragm respiration in the theta LFP cycles of the rats' OB. In this case the coefficients were: Rat 1, β_1_ = 0.78, β_0_ = 0.029, *r* = 0.64; Rat 2, β_1_ = 0.76, β_0_ = 0.032, *r* = 0.62; Rat 3, β_1_ = 0.91, β_0_ = 0.016, *r* = 0.86; Rat 4, β_1_ = 0.93, β_0_ = 0.011, *r* = 0.84. (see Figure 4D, red-dots scatter plots)

We also analyzed the low frequency (1–2 Hz) respiratory cycles from Rat 3. These slower cycles showed an EMG-LFP delay of about 200 ms, longer than the rest of the rats and himself at higher (4–12 Hz) respiratory frequencies (Figure 5). Regardless of this difference, it was clear from the data that the LFP oscillations were following the respiratory cycles at these low frequencies (Figure 5A) as well as they did at higher respiratory rates. The relationship between respiration and the LFP is also clear in inhalation-triggered LFP means, both in low (Figure 5B) and high (Figure 5C) respiratory frequencies.

We analyzed a previously described relationship between low and high frequencies within the OB LFP. Gamma activity bursts (40–100 Hz) in the OB LFP are reported to occur phase-locked to theta oscillations (Freeman, 1976; Rojas-Líbano and Kay, 2008). This can be quantified using phase-amplitude coupling (PAC) measures such as the modulation index (Tort et al., 2010) between the user-defined low and high frequencies. We computed these coupling measures for the rats' LFP and found a consistent PAC for all the rats with LFP and EMG data (Figure 6). Gamma activity, which in our recordings was 80–100 Hz for the most part, was consistently modulated by theta phase, increasing in amplitude in the second half (from phase = 0 to phase = π) of each theta cycle.

During the recording sessions, all rats alternated between two main behavioral modes: at first, they actively explored the cage, covering most of its area. Occasionally they stopped and sniffed with the tip of the nose close to the bedding, the walls or the air space above, and then resumed body movement. In the second behavioral mode, rats nose poked at a hole in one of cage walls and stayed still with the nose protruding a little to the outside of the cage, sniffing. The wall hole is the place where the rat normally finds the drinking tube of a water bottle, which was removed for the recording session. In general, rats spent 30% of the session in the nose-poke mode and the remaining 70% in the exploratory mode (see Figure 7A).

Respiratory behavior was considerably modified as a function of the behavioral modes. This is illustrated in Figure 7. In the exploratory mode, respiratory frequency varied between 4 and 12 Hz. In contrast, during nose-poke behavior it remained between 5.5 and 6.5 Hz, with very little variation throughout the period (Figure 7B). LFP power decreased drastically during nose-poke, both in theta and gamma ranges (Figures 7C,D). Coupling between LFP and EMG, however, remained high in both behavioral modes (Figure 7E). It is interesting to note that the reduction in LFP power seen during nosepoke periods was not the result of reduced inhalation strength, as rsEMG amplitude did not decrease compared to exploration periods (Figure 7F).

## Discussion

The diaphragm, one of the main muscle targets of the neuronal respiratory network, and the olfactory bulb, the first brain structure to receive input from olfactory sensory neurons, were recorded during free behavior of rats exploring their home cages. We found that rats spontaneously displayed a full range of respiratory frequencies from 1 to 12 Hz and could switch rapidly between frequencies, in 1–2 respiratory cycles (Figures 1–2). The higher end of this frequency band is also associated with sniffing behavior seen in learning and odor discrimination contexts (Rojas-Líbano and Kay, 2012). We found a close relationship between motor respiratory activity and sensory LFPs in the OB, expressed as spectral coherence between the signals (Figure 3) and as a correlation of the cycle durations measured from both signals (Figure 4). Our results indicate a delay of about 80 ms from inhalation onset at the diaphragm and signal arrival in the OB (Figure 4), for sniffing (4–12 Hz) respiratory frequencies, and a delay of 200 ms for lower (<1.5 Hz) respiratory frequencies. We found as well a consistent LFP phase-amplitude coupling in the OB between respiratory-related frequencies (1–15 Hz) and gamma frequencies (40–100 Hz), as shown in Figure 6. In addition, we found a regular association between the behavioral modes exhibited in our setup and specific motor and sensory patterns of the recorded structures.

A sequence of several events is known to translate the diaphragm motor output to the respiratory rhythm in the OB. As the diaphragm contracts, air is drawn into the nose and generates a pressure wave that mechanically stimulates the OSNs and brings odorant molecules into contact with the mucosa. The OSNs appear to respond to both odorant binding at the receptors and mechanical stimulation (Grosmaitre et al., 2007), which in turn sends a volley of activity through the olfactory nerve that reaches the OB. This volley is translated by intraglomerular circuits and read by electrodes as a respiratory-linked oscillation of the LFP. If sensory drive by the olfactory nerve is interrupted by tracheotomy, naris closure or nerve transection, OB theta oscillations are almost completely abolished (Domino and Ueki, 1960; Gault and Leaton, 1963; Gray and Skinner, 1988; Courtiol et al., 2011a). Changes in respiratory cycles through the manipulation of pressure changes in the nasopharynx, change the corresponding theta frequency in the OB (Fontanini et al., 2003; Courtiol et al., 2011b; Esclassan et al., 2012).

Respiratory modulation of OB neural activity also occurs at the single neuron level (Walsh, 1956; Macrides and Chorover, 1972; Onoda and Mori, 1980; Ravel et al., 1987), with most reports recording from mitral/tufted cells. In this context, modulation means that the spiking probability of a fraction of the recorded cells is a function of the respiratory phase. Whereas some studies report that a little modulation survives after bypassing nasal breathing (Ravel et al., 1987), some others report complete abolition of modulation in conditions of no nasal breathing (Phillips et al., 2012). At any rate, it seems that the majority of the modulation comes from nasal neural input and a relatively small fraction from centrifugal inputs to the OB.

Despite reference to the respiratory rhythm in the literature over many decades, it was not known if the respiratory-related theta oscillation in the OB would follow the changing respiratory rhythms in the normal range of frequencies displayed spontaneously by behaving rats. Previous studies that had addressed this question were done in anesthetized animals that breathe at very low rates. If the rhythms are tightly linked at all frequencies, then this indicates that the OB circuit has access to and incorporates the motor program at all times. In all the rats we recorded we were able to show not only a spectral coherence between the OB LFP and the diaphragm EMG, but also a cycle-by-cycle correlation between the duration of respiratory and theta LFP cycles, with the LFP cycle lagging respiration, for all the respiratory frequencies exhibited by the rats, which in our case had the range 1–15 Hz. We found a consistent delay estimate of approximately 80 ms from the diaphragm (motor output) signal to the OB theta band LFP (cortical) signal at sniffing frequencies. It takes on the order of 20–40 ms for the diaphragm contraction to begin changing the airflow in the nose (estimated from Katz et al., 1962); we therefore conclude that the remainder of the 80 ms delay between the diaphragm signal and the OB theta oscillation is due to sorption and diffusion of odorants, transduction of odorant binding and mechanical stimulation by ORNs, transduction delays of the sensory signal and the time it takes for the target neural circuits in the OB to respond to the sensory volley.

Another prominent feature of the OB theta oscillation previously described is the phase-amplitude coupling (PAC) between the theta (1–15 Hz in the rat) and the faster gamma (40–100 Hz in the rat) oscillations. The PAC is characterized by bursts of gamma that occur phase-locked to the second half (from phase = 0 to phase = π) of the theta cycle. Extensive work done in anesthetized rats has confirmed this PAC, at least under urethane anesthesia (Buonviso et al., 2003; Cenier et al., 2009). In the awake rat, it is customary to assume that the PAC is always present, although only example traces are available in the literature (Freeman, 1976; Kay et al., 1996; Martin et al., 2004; Courtiol et al., 2011b). Again, in the entire range of frequencies exhibited by our rats we were able to detect this PAC, quantified by the Modulation Index (MI) created by Tort et al. (2010). The MI analysis of the OB LFPs showed that the bursts of gamma activity (40–100 Hz in our rats) occurred during the second half of the theta cycle (Figure 6) irrespective of the theta frequency. A recent report shows that this PAC is abolished during sleep (Manabe and Mori, 2013), where the “nested” gamma oscillations, as the authors refer to, could not be detected either during short-wave sleep or during rapid-eye movement sleep. These authors also report high and low frequencies for gamma oscillations, peaking around 80 and 60 Hz respectively and appearing sequentially and link these to a recent report about firing probabilities of tufted vs. mitral cells at the inhalation to exhalation transition and during late exhalation, respectively (Fukunaga et al., 2012). In our sessions, we did not see much low-frequency gamma, with most activity located between 80 and 100 Hz. However, we have reported previously the occurrence of these two types of gamma (Kay, 2003; Rojas-Líbano and Kay, 2008), especially during grooming, waiting or immobility behaviors. The fact that we did not report them here is mainly related to the types of spontaneous behaviors displayed by our rats within the span of the 10–15 min of our recordings, which was for the most part active exploration. In addition, in our rats we saw little or no beta LFP activity (20–35 Hz), which has been reported to occur mainly in response to high volatility chemicals delivered close to the animal's nostrils or as a result of operant associative olfactory learning (Ravel et al., 2003; Lowry and Kay, 2007; Martin et al., 2007; Aylwin et al., 2009).

We also report a spontaneous switch between two behavioral modes, which we found in all recorded rats. They either explored the cage, moving around it actively sniffing, or they nose-poked at a hole in the cage and stayed there, locking their respiration frequency at about 6 Hz. The switch between these respiration/behavioral modes was reflected clearly in the OB LFP, with marked changes in power at all frequencies. In turn, spectral coherence between respiration and LFP was not affected by these modes, reflecting again the capabilities of the system to maintain coupling at different frequencies (Figure 7).

Sniffing strategies show many connections to behaviors associated with learning and remembering odors. The sniffing rhythm itself, although it overlaps in frequency with the hippocampal theta rhythm implicated in learning and memory processes, is not normally coherent with the hippocampal rhythm. This means that there is no consistent phase relationship between the signals. However, in some learning conditions the rhythms do become coherent. One study showed that when rats learned a reward contingency reversal, sniffing showed coherence with the hippocampal theta rhythm which decayed with learning (Macrides et al., 1982). The opposite effect was shown during learning and performance of a difficult go/no-go task in which rats had to keep track of timing (Kay, 2005). In this condition OB and hippocampal theta rhythm coherence was positively correlated with performance.

Rats also adjust their sniffs as they learn; recent work from our laboratory has shown that rats use variable sniff flow strategies to detect odors dependent on their sorptive properties (Rojas-Líbano and Kay, 2012). As rats learn to discriminate odors, they increase sampling duration and use longer inhalations for low than high sorption odorants, but upon reaching criterion performance they produce lower flow sniffs for low sorption than for high sorption odorants. The current study now shows that the theta rhythm in the OB can be used to study *respiratory frequency* on a cycle-by-cycle basis and to track the sensorimotor representation within the neural circuit.

### Conflict of interest statement

The authors declare that the research was conducted in the absence of any commercial or financial relationships that could be construed as a potential conflict of interest.
